# Salivary and serum leptin levels in oral lichen planus patients. A case-control study

**DOI:** 10.1038/s41405-025-00358-0

**Published:** 2025-08-05

**Authors:** Bahaa Mahmoud Fawzy El Nomrosy, Weam Ahmed Maher Rashawn, Olfat Gamil Shaker

**Affiliations:** 1 B.D.S. Misr University for Science and Technology (2015), Giza, Egypt; 2 Diagnosis & Periodontology—Faculty of Dentistry, Cairo University, Cairo, Egypt; 3 Medical Biochemistry and Molecular Biology—Faculty of Medicine, Cairo University, Cairo, Egypt

**Keywords:** Gum disease, Oral medicine

## Abstract

**Background:**

Lichen planus (LP) is a chronic inflammatory disease. Oral lichen planus (OLP) is a chronic inflammatory disease that affects the oral mucosa. Different clinical types of oral lichen planus can be diagnosed based on clinical presentation and histopathological features.

**Aim:**

The present study aimed to assess the potential of salivary and serum leptin in the diagnosis of OLP.

**Materials & Methods:**

The study was conducted on 78 cases (i.e., 39 cases per group) with OLP. The subjects were systematically free. Salivary and serum leptin concentrations from patients exhibiting a classic OLP and from healthy controls were assessed.

**Results:**

Salivary and serum leptin levels are higher in the OLP group. The participants in the study are older than those in the control group; therefore, the age difference between the two groups does not affect the study’s results.

**Conclusion:**

Salivary and serum leptin levels in OLP patients were higher than in healthy control subjects, suggesting a possible role in the process of OLP pathogenesis.

## Introduction

LP is a common condition affecting stratified squamous epithelia. It is a chronic, mucocutaneous, and immunological disorder characterized by a range of clinical manifestations. The oral mucosa is frequently involved, and in some cases, it may be the sole site of involvement [1, 2]. OLP can present in six distinct clinical forms: reticular, papular, plaque-like, atrophic (erythematous), erosive-ulcerative, and bullous-erosive [3]. The buccal mucosa is the most affected site, followed by the lingual, gingival, and labial mucosa [4]. A hallmark feature of OLP is the presence of Wickham striae, white hyperkeratotic papules that create a lace-like appearance in the lesions [5].

The immunopathogenesis of OLP is believed to involve multiple mechanisms, including antigen-specific cell-mediated immune responses, non-specific processes, autoimmune activity, and humoral immunity [2]. Histopathological features characteristic of OLP include hyperkeratosis, acanthosis or epithelial atrophy, basal keratinocyte liquefaction, and a dense, band-like lymphocytic infiltrate in the superficial lamina propria. Additionally, epithelial ridges often exhibit a saw-tooth appearance. Amorphous eosinophilic deposits, known as Civatte bodies, are present in both the basal cell layer and superficial lamina propria [6].

Leptin, a polypeptide hormone secreted by white adipose tissue, plays a role in immune responses and has been linked to dyslipidemia in LP [7]. Studies have consistently demonstrated a correlation between elevated levels of leptin, increased body fat, and higher body mass index (BMI) [7]. Leptin also contributes to cellular immune responses and the promotion of autoimmunity. The objective of the present study was to evaluate the diagnostic potential of salivary and serum leptin levels in OLP.

## Methods

### Study design

The present study is a case-control study.

### Study setting

The study included 39 patients with OLP and 39 control subjects, selected from the outpatient clinic of the Oral Medicine, Periodontology, and Oral Diagnosis Department at Cairo University’s Faculty of Dentistry. The study protocol was registered on the registry site clinicaltrials.gov (NCT06078579). The study was conducted from December 2023 to September 2024. This study follows the CONSORT statement and was performed according to the Declaration of Helsinki.

### Ethical aspect

The Ethical Committee, Faculty of Dentistry, Cairo University, certified the study and all accompanying documentation for research and ethical approval before the commencement of the study, in compliance with local rule number 20/7/23. The researchers explained the study’s purpose and methodology to the participants. After reading the written informed consent, all participants gave their approval and signed it.

### Sample size calculation

Power analysis was conducted to ensure sufficient statistical power to use a two-sided statistical test of the null hypothesis that there is no difference between the tested groups regarding salivary and serum leptin levels. With an alpha (α) level of 0.05 (5%), beta (β) level of 0.2 (80% power), and effect size (d) of 0.736 based on previous study results [7], the anticipated sample size (n) was (62) cases (31 each group). To account for drop-out during follow-up intervals, the researchers increased the sample size by 25% to reach 78 patients (39 for each group). They calculated the sample size using G*Power 3.1.9.7.

### Subject selection

#### Inclusion criteria

- Both sexes have an age range of 30–70.
- Symptomatic OLP has been clinically diagnosed and histologically confirmed.
- Participants who sign a formal consent form after receiving complete information about the study.

#### Exclusion criteria

- Treatment with a systemic or locally delivered medicine within three months prior to research start. may alter tissue expression of inflammatory mediators.
- Patients who are now taking or have recently discontinued using NSAIDs may see changes in tissue expression of inflammatory mediators.
- Patients diagnosed with malignant tumors.
- Women who are pregnant or breastfeeding.
- Category includes inmates, the mentally sick, elderly, and etc.

### Clinical examination

#### Group I (OLP patients)

Group I patients were diagnosed with various clinical forms of OLP. Diagnosis of oral lichen planus was established based on the clinical and histopathological criteria recommended by the American Academy of Oral and Maxillofacial Pathology [8]. Clinically, lesions presented bilaterally with typical reticular white striae, often accompanied by atrophic or erosive areas. Histopathologically, specimens showed a band-like lymphocytic infiltrate in the superficial connective tissue with basal cell degeneration [8].

Oral lesions were categorized as reticular, atrophic, and bullous-erosive. The patients underwent a clinical examination using a spotlight and magnifying glass for mouth lesions and natural light for skin lesions. The finding of Wickham’s striae provided definitive confirmation for the clinical diagnosis. Stretching the mucosa on the surface would highlight these striae, which cannot be removed by rubbing. The criteria of [9, 10] were used to evaluate the clinical symptoms. They determined the degree of lesions and symptoms in OLP patients as follows: 5 = white striae with erosion ≥1 cm^2^. 4 = white striae with erosion < 1 cm^2^. 3 = white striae with atrophy ≥ 1 cm^2^. 2 = white striae with atrophy ≤ 1 cm^2^. 1 = white striae only; and 0 = no lesion. The severity of the symptoms was evaluated [11]. The researchers evaluated the severity of symptoms in participants diagnosed OLP as follows: 3 = severe symptoms, 2 = moderate symptoms, 1 = mild symptoms, and 0 = absence of symptoms.

#### Group II (control group)

Using the Medical Complexity Status Classification and Protocol.

#### Inclusion criteria

The control group consisted of healthy individuals without a history or clinical signs of oral lichen planus or other oral mucosal lesions. Controls were selected to ensure they had no systemic conditions, were not on immunosuppressive therapy, and had no habits such as tobacco use that could influence oral mucosa health.

#### Exclusion criteria

Any history of autoimmune or inflammatory diseases, recent medication use that could affect oral tissues, and presence of dental restorations known to cause lichenoid reactions (e.g., amalgam fillings).

### Pretreatment evaluation

During the initial assessment, the subject’s and/or patient’s name, age, gender, date of initial diagnosis of their lesions, as well as their medical and dental history, were collected. The researchers obtained baseline data and conducted clinical oral examinations. They documented all demographic, clinical, and laboratory data photographically for each patient in the two study groups, including the extent and location of mucosal/cutaneous lesions, illness severity scores, and the biopsy site. They then verified the diagnosis through biopsy.

### Biopsy procedures

Surgical double wedge oral mucosal incisional biopsy specimens of approximately 5 mm were taken from the oral mucosa of Group I patients after performing ring anesthesia (administration of ring block anesthesia). The biopsies were fixed in formalin and were processed into paraffin-embedded tissue blocks according to routine practice. Hematoxylin and eosin–stained slides (6 µm) were used for routine diagnosis. The slides were reexamined to confirm the diagnosis of OLP before including the samples for the study.

### Outcome assessment

#### Quantitation of Human Leptin in saliva and serum

The levels of leptin in the saliva and serum of patients with LP, as well as healthy controls, were measured using an Enzyme-Linked Immunosorbent Assay (ELISA) kit provided by Bioassay Technology Laboratory (Shanghai, China), Cat. No E1559Hu. The kit Sensitivity was 0.021 ng/ml.

#### Assay principle

This kit is based on the ELISA method. The assay plate is pre-coated with human leptin antibodies. When the sample is added, any leptin present binds to these antibodies. Subsequently, a biotinylated human leptin antibody is introduced, which attaches to the leptin in the sample. Streptavidin-HRP is then added, binding to the biotinylated leptin antibody. After an incubation period, unbound Streptavidin-HRP is removed through a washing step. A substrate solution is added, producing a color change proportional to the leptin concentration. The reaction is terminated by adding an acidic stop solution, and the absorbance is measured at 450 nm.

### Assay procedure

1. **Preparation of Reagents and Standards:** All reagents and standard solutions were prepared according to the instructions provided. All reagents were brought to room temperature before use, and the assay was conducted at room temperature.
2. **Addition of Standards:** 50 μL of the standard solution was added to the standard wells.
3. **Addition of Samples:** 40 μL of the sample was added to the sample wells.10 μL of anti-leptin antibody was then added to the sample wells. 50 μL of Streptavidin-HRP was added to both the sample and standard wells (excluding the blank control well). The contents were thoroughly mixed, the plate was sealed with a cover and then incubated for 60 min at 37 °C.
4. **Washing Step:** The sealer was removed, and the plate was washed five times using the wash buffer. After washing, the plate was blotted dry using paper towels or other absorbent material.
5. **Addition of Substrate Solutions:** 50: μL of substrate solution A was added to each well, followed by 50 μL of substrate solution B. The plate was covered with a fresh sealer and incubated in the dark at 37 °C for 10 min.
6. **Stop Reaction:** 50: μL of stop solution was added to each well, causing the color to change from blue to yellow.
7. **Measurement of Optical Density (OD):** The OD of each well was immediately measured using a microplate reader set to 450 nm. This measurement was completed within 10 min of adding the stop solution.

### Statistical analysis

Categorical data were expressed as frequencies and percentages and analyzed using Fisher’s exact test. Numerical data were summarized as mean, standard deviation (SD), median, and interquartile range (IQR) values. The normality of the data was assessed by examining the distribution and using Shapiro-Wilk’s test. Age data followed a normal distribution and were compared using an independent t-test. Non-parametric data were analyzed using the Mann–Whitney U test. Correlations were evaluated using Spearman’s rank-order correlation coefficient. Receiver Operating Characteristic (ROC) curves were constructed to assess the diagnostic accuracy of various markers, with the optimal cut-off value determined using the Youden index. Comparisons of ROC curves were conducted using DeLong’s test. A significant level of *p* < 0.05 was applied for all statistical tests. Statistical analyses were performed using R statistical software version 4.4.1 for Windows.

## Results

### Demographic data

Intergroup comparison and summary statistics for demographic data are presented in (Table 1). The study was conducted in 78 cases (i.e., 39 cases per group). There were 13 males and 26 females in the OLP group, with a mean age of (50.77 ± 13.37) years. In the control group, there were 18 males and 21 females with a mean age of (34.51 ± 12.53). The mean BMI index for the OLP group was (28.47 ± 3.95) (Kg/m^2^), and for the control group, it was (28.77 ± 3.32) (Kg/m^2^). There was no significant difference between both groups regarding sex (*p* = 0.355) and BMI index (*p* = 0.179). However, the cases in the OLP group had significantly higher ages than the controls (*p* < 0.001).

**Table 1:** Intergroup comparison and summary statistics for demographic data.

Parameter		OLP	Control	*p* value
Sex [n (%)]	Male	13 (33.33%)	18 (46.15%)	0.355^ns^
	Female	26 (66.67%)	21 (53.85%)	
Age (years)	Mean ± SD	50.77 ± 13.37	34.51 ± 12.53	<0.001*
	Median (IQR)	55.00 (41.5–60.0)	31.00 (24.5–39.5)	
BMI (Kg/m^2^)	Mean ± SD	28.47 ± 3.95	28.77 ± 3.32	0.179^ns^
	Median (IQR)	27.70 (26.4–30.05)	28.80 (27.6–30.15)	

^*^Significant, ns not significant.

### ANCOVA analysis for adjustment for age as a covariate

To address the age difference between groups and ensure the reliability of our findings, we performed an analysis of covariance (ANCOVA) with age as a covariate. For salivary leptin, the group effect remained significant with F (1, 75) = 53.90, *p* < 0.001. The adjusted mean salivary leptin level was 14.7 ± 0.8 ng/mL for the OLP group and 5.5 ± 0.8 ng/mL for controls; the effect of age itself was not statistically significant (serum leptin: *p* = 0.29; salivary leptin: *p* = 0.08) (Table 2).

**Table 2:** ANCOVA analysis for salivary leptin levels after adjustment of age as a covariate.

Group	*N*	Adjusted Mean ± SD (ng/mL)	F (1,75)	*p* value
OLP	39	14.7 0.8	53.90	<0.001
Control	39	5.5 ± 0.8		

Analysis adjusted for age as a covariate. Age was not statistically significant (*p* = 0.08).

To address the age difference between groups and ensure the reliability of our findings, we performed an analysis of covariance (ANCOVA) with age as a covariate. For serum leptin, the effect of group (OLP vs. control) was significant with F (1, 75) = 34.35, *p* < 0.001. The age-adjusted mean serum leptin concentration was 12.8 ± 0.9 ng/mL in the OLP group versus 4.6 ± 0.9 ng/mL in the control group, indicating substantially higher leptin levels in OLP patients (Table 3).

**Table 3:** ANCOVA analysis for serum leptin levels after adjustment of age as a covariate.

Group	N	Adjusted Mean ± SD (ng/mL)	F (1,75)	*p*-value
OLP	39	12.8 ± 0.9	34.35	< 0.001
Control	39	4.6 ± 0.9		

Analysis adjusted for age as a covariate. Age was not statistically significant (*p* = 0.29).

### OLP characteristics

**Fig. 1:**
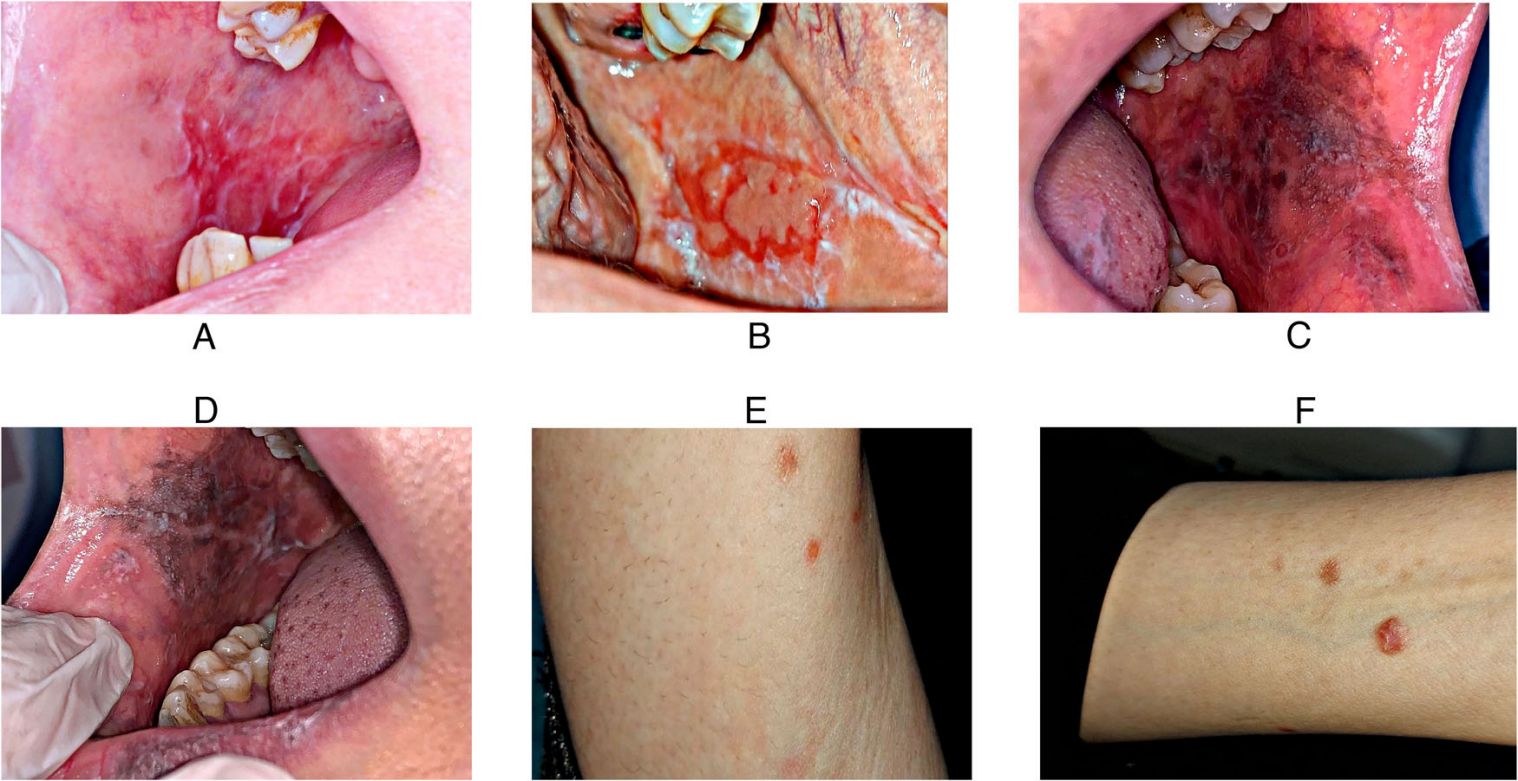
Clinical presentation of OLP lesions. **A** a patient suffering from bullous erosive and reticular oral lichen planus (the right buccal mucosa); **B** The left buccal mucosa of the patient shows erosive and reticular OLP; **C** the patient’s left buccal mucosa shows reticular form or OLP; **D** Left buccal mucosa shows the erosive and reticular form of OLP; **E** Maculopapular rash on the right arm of the patient indicates she is also suffering from mucocutaneous OLP; **F** Left arm of the patient also showing maculopapular rash of mucocutaneous OLP.

OLP characteristics are presented in (Table 4). Among the 39 cases, the most common OLP type was bullous-erosive (69.23%), followed by reticular (30.77%) and atrophic (5.13%). All patients were medically free. Extra-oral lesions were absent in most cases (76.92%), with only (23.08%) having such lesions (Fig. 1). Regarding lesion size, most cases (56.41%) had white striae with an erosive area larger than 1 cm². In comparison (25.64%) had mild white striae without erythematous or erosive areas, (12.82%) had white striae with an erosive area smaller than 1 cm², and only (5.13%) showed white striae with an atrophic area larger than 1 cm². Lesion severity was reported as severe in (64.10%) of cases, mild in (28.21%), and moderate in (7.69%).

**Table 4:** OLP characteristics.

Parameter		Value
Type of OLP [n (%)]	Reticular	12 (30.77%)
	Atrophic	2 (5.13%)
	Bullous-Erosive	27 (69.23%)
Medically compromised [n (%)]	No	39 (100.0%)
	Yes	0 (0.0%)
Extra-oral lesions [n (%)]	No	30 (76.92%)
	Yes	9 (23.08%)
Lesion size [n (%)]	Mild white striae without erythematous or erosive	10 (25.64%)
	White striae with atrophic area < 1 cm^2^	0 (0.0%)
	White striae with atrophic area ≥ 1 cm^2^	2 (5.13%)
	white striae with erosive area < 1 cm^2^	5 (12.82%)
	white striae with erosive area ≥ 1 cm^2^	22 (56.41%)
Lesion severity [n (%)]	Mild	11 (28.21%)
	Moderate	3 (7.69%)
	Severe	25 (64.10%)
Pain score	Mean ± SD	5.74 ± 3.06
	Median (IQR)	6.00 (5.00)

### Salivary leptin

Intergroup comparison and summary statistics for salivary leptin (ng/ml) are presented in (Table 5). Values measured in the OLP group were significantly higher than those of the controls (*p* < 0.001).

**Table 5:** Intergroup comparison and summary statistics for salivary leptin (ng/ml) and serum leptin (ng/ml).

Measurement	Group		*P* value
	OLP	Control	
Salivary Leptin (ng/ml)			
Mean ± SD	15.31 ± 6.59	4.90 ± 1.26	<0.001*
Serum Leptin (ng/ml)			
Mean ± SD	13.23 ± 7.29	4.20 ± 1.23	<0.001*

*Significant (*p* < 0.05).

### Serum leptin

Intergroup comparison and summary statistics for serum leptin (ng/ml) are presented in (Table 5). Values measured in the OLP group were significantly higher than those of the controls (*p* < 0.001).

### Correlation between salivary and serum leptin

**Fig. 2:**
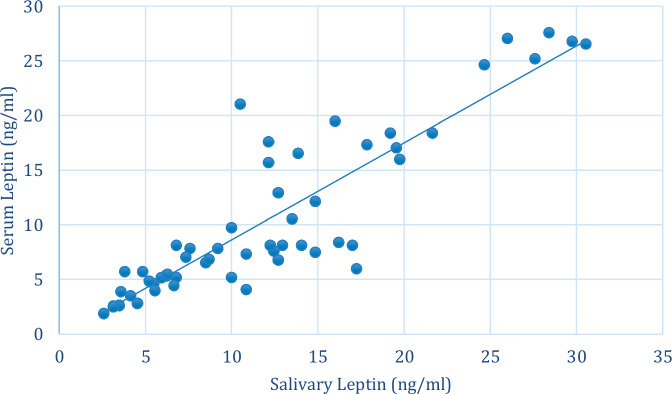
Scatter plot showing the correlation between salivary and serum leptin.

The correlation between salivary and serum leptin is presented in (Table 6) and (Fig. 2). There was a strong positive correlation between salivary and serum leptin that was statistically significant (*r*_*s*_ = 0.890, *p* < 0.001).

**Table 6:** Intergroup comparison and summary statistics for salivary and serum leptin.

Variables	Correlation coefficient (95% CI)	*P* value
Salivary and serum Leptin	0.890 (0.832:0.928)	<0.001*

*CI* Confidence interval, *significant (*p* < 0.05).

### Association between OLP type and salivary leptin

The association between OLP type and salivary leptin (ng/ml) is presented in (Table 7). The association was statistically significant, with cases affected having significantly higher values than the controls (*p* < 0.001). However, the differences between different OLP types were not statistically significant.

**Table 7:** Association between OLP type and salivary leptin (ng/ml) and serum leptin (ng/ml).

Measurement					*P* value
	Reticular	Bullous-Erosive	Atrophic	Control	
Salivary Leptin (ng/ml)	14.76 ± 7.55^A^	15.45 ± 6.45^A^	16.80 ± 4.17^A^	4.90 ± 1.26^B^	<0.001*
Serum Leptin (ng/ml)	13.83 ± 7.46^A^	12.70 ± 7.58^A^	16.27 ± 0.39^A^	4.20 ± 1.23^B^	<0.001*

Values with different superscript letters within the same horizontal row are significantly different *Significant (*p* < 0.05).

### Association between OLP type and serum leptin

The association between OLP type and serum leptin (ng/ml) is presented in (Table 7). The association was statistically significant, with cases affected having significantly higher values than the controls (*p* < 0.001). However, the differences between different OLP types were not statistically significant.

### Diagnostic accuracy

ROC curve analysis for the diagnostic accuracy of salivary and serum leptin is presented in (Table 8). Salivary leptin had higher sensitivity and negative predictive value (NPV) than serum leptin, indicating it is more effective in detecting individuals with OLP and ensuring that those with a negative result are unlikely to have the disease. Both markers were precise with (100%) specificity and positive predictive values (PPVs), confirming a strong ability to identify OLP accurately when a positive result is obtained, as there were no false positives. The AUC value for salivary leptin (1) was higher than that of serum leptin (0.971). However, the difference was not statistically significant (*p* = 0.110).

**Table 8:** ROC curve analysis for salivary and serum leptin.

Parameter	Value [95%CI]		*P* value
	Salivary leptin	Serum leptin	
Sensitivity	100% (100%:100%)	92.31% (82.05%:100%)	0.110 ^ns^
Specificity	100% (100%:100%)	100% (100%:100%)	
Cut off point	>=6.80	>=6.00	
Negative predictive value (PPV)	100% (100%:100%)	92.86% (84.78%:100%)	
Positive predictive value (NPV)	100% (100%:100%)	100% (100%:100%)	
Area under the curve (AUC)	1 (1:1)	0.971 (0.936:1)	

*CI* confidence interval, *ns* not significant.

## Discussion

OLP is a chronic inflammatory condition of the oral mucosa, affecting 1–2% of the population. It is more common in middle-aged and older women, with a female-to-male ratio of 1.5:1 [12, 13].

Leptin, a polypeptide hormone secreted by white adipose tissue, has been shown to increase in individuals with higher body mass index (BMI) and total body fat percentage. It also plays a role in the cellular immune response and the promotion of autoimmunity [7]. Leptin may have a potential role in the pathophysiology of LP; however, data on leptin levels in LP patients remain limited.

The present study aimed to evaluate the diagnostic potential of salivary and serum leptin in OLP. The study included 78 participants, divided into two groups: **Group I**, comprising 39 patients diagnosed with active OLP. **Group II:** 39 healthy individuals serving as controls.

Both groups were systemically healthy, as assessed using the Medical Complexity Status Classification and Protocol ^8^. Patients in Group I exhibited various clinical forms of OLP and were selected based on specific criteria [8]. The diagnosis was confirmed histopathologically via biopsy.

The findings of this study are consistent with previous research regarding age and gender distribution, showing a higher prevalence of OLP in females and individuals over 40 years of age [14, 15]. In the OLP group, 13 patients (33.33%) were male, and 26 (66.67%) were female, with a mean age of 50.77 ± 13.37 years. In the control group, 18 participants (46.15%) were male, and 21 (53.85%) were female, with a mean age of 34.51 ± 12.53 years. The mean BMI was 28.47 ± 3.95 kg/m² in the OLP group and 28.77 ± 3.32 kg/m² in the control group (Table 1).

Statistical analysis revealed no significant differences between the two groups in terms of sex (*p* = 0.355) or BMI (*p* = 0.179). However, the OLP group was significantly older than the control group (*p* < 0.001). Although serum leptin levels are known to decline with age, the decline is more pronounced in women [16]. The higher salivary and serum leptin levels observed in the OLP group were attributed to the disease itself. The age difference between the groups did not influence the study’s outcomes (Table 1).

The researchers analyzed covariance (ANCOVA) with age as a covariate to control for age differences between groups. The analysis showed that group differences in serum and salivary leptin levels remained highly significant after adjusting for age (serum: F (1,75) = 34.35, *p* < 0.001; saliva: F (1,75) = 53.90, *p* < 0.001). Age itself was not a significant predictor in either model, indicating that elevated leptin levels in OLP patients are independent of age (Tables 2–3).

OLP characteristics: In the 39 cases, the most common OLP type was bullous-erosive 27 (69.23%), followed by reticular 12 (30.77%) and atrophic 2 (5.13%). Extra-oral lesions were absent in most cases (30 cases, 76.92%), with only 9 cases (23.08%) having such lesions (Table 4).

Regarding lesion size, in most cases, 22 (56.41%) had white striae with an erosive area larger than 1 cm². In comparison, 10 (25.64%) of cases had mild white striae without erythematous or erosive areas, and 5 cases (12.82%) had white striae with an erosive area smaller than 1 cm². Only 5 (5.13%) of cases showed white striae with an atrophic area larger than 1 cm². Lesion severity was reported as severe in 25 (64.10%) of cases, mild in 11 (28.21%) of cases, and moderate in 3 (7.69%) of cases (Table 4).

The intergroup comparison of salivary leptin levels that were measured in the OLP group was significantly higher than those of the controls (*p* < 0.001). The mean value of Salivary Leptin levels in the OLP group was 15.31 ± 6.59, whereas the mean value of salivary leptin levels in the control group was 4.90 ± 1.26. These results suggest a role of salivary leptin in the pathogenesis of OLP and a possible role as a diagnostic biomarker (Table 5).

Serum leptin levels in the OLP group were significantly higher than those in the control group (*p* < 0.001). The mean serum leptin level in the OLP group was 13.23 ± 7.29 ng/mL, compared to 4.20 ± 1.23 ng/mL in the control group. Similar findings were reported, supporting the role of serum leptin in the pathogenesis of OLP and its potential as a diagnostic biomarker (Table 5) [16].

A strong positive correlation between salivary and serum leptin levels was observed, which was statistically significant (*r*_*s*_ = 0.890, *p* < 0.001). These findings suggest that salivary and serum leptin contribute similarly to the pathogenesis of OLP, highlighting their potential as diagnostic biomarkers for this condition (Table 6).

The association between OLP type and salivary leptin levels was statistically significant, with OLP cases showing markedly higher levels than the control group (*p* < 0.001). However, differences in salivary leptin levels among the various OLP types were not statistically significant. The mean salivary leptin levels were as follows: Reticular OLP: 14.76 ± 7.55 ng/ml, bullous-erosive OLP: 15.45 ± 6.45 ng/ml, atrophic OLP: 16.80 ± 4.17 ng/ml, control group: 4.90 ± 1.26 ng/ml. These findings underscore the role of leptin in the pathogenesis of OLP and its potential diagnostic significance (Table 7).

The association between OLP type and serum leptin was statistically significant, with cases affected having significantly higher values than the controls (*p* < 0.001). However, the differences between different OLP types were not statistically significant. The mean value of reticular OLP serum leptin was 13.83 ± 7.46 ng/ml, whereas the mean value of bullous erosive OLP serum leptin was 12.70 ± 7.58 ng/ml. In contrast, the mean value of atrophic OLP serum leptin was 16.27 ± 0.39 ng/ml. In contrast, the mean value of the control group serum leptin was 4.20 ± 1.23 ng/ml, suggesting a role for the serum leptin in the pathogenesis of OLP (Table 7).

The diagnostic accuracy of salivary and serum leptin for detecting OLP was evaluated using ROC curve analysis. Salivary leptin demonstrated superior sensitivity (100%) and negative predictive value (NPV) (100%) compared to serum leptin, which showed a sensitivity of 92.31% and an NPV of 92.86%. These findings suggest that salivary leptin is more effective in identifying individuals with OLP and in ruling out the disease when test results are negative. Both markers exhibited excellent specificity (100%) and positive predictive values (PPVs), underscoring their substantial capacity to diagnose OLP, with no false-positive results observed accurately. The area under the curve (AUC) for salivary leptin was 1.0, slightly higher than the AUC for serum leptin (0.971). However, this difference was not statistically significant (*p* = 0.110) (Table 8).

The present study revealed significantly higher serum and salivary leptin levels in patients with OLP compared to healthy controls, even after adjusting for age using analysis of covariance (ANCOVA). These findings support the hypothesis that leptin may play a role in the pathogenesis of OLP, potentially through its known pro-inflammatory and immune-modulatory functions.

The elevated leptin levels in both serum and saliva are consistent with previous studies indicating increased leptin expression in other autoimmune or chronic inflammatory diseases. These findings suggest that OLP, being a chronic inflammatory mucosal condition, may share similar inflammatory mechanisms.

Notably, a subset of our patients presented with both oral and cutaneous lichen planus. It supports the concept that lichen planus is a multisite mucocutaneous disease, and the consistent elevation of leptin levels in these patients may indicate a broader systemic alteration in immune regulation. Previous studies on cutaneous lichen planus (CLP) have also reported altered leptin profiles, further reinforcing this possible link.

The current study demonstrated elevated leptin levels in patients with OLP compared to healthy controls. These findings are in agreement with Aryanian et al. [17], who reported increased leptin levels in patients with CLP and suggested a link with metabolic syndrome components. Although their study focused on systemic associations and included cutaneous forms of LP, while ours focused on the oral variant, the consistent elevation in leptin levels across both forms suggests a possible shared inflammatory or metabolic pathway. Further comparative studies are needed to clarify whether these findings reflect a general feature of lichen planus or are specific to certain subtypes.

Additionally, LP and OLP are chronic inflammatory conditions that share similar immunopathogenic mechanisms, primarily involving T-cell-mediated immune responses. However, their clinical presentations differ based on the site of involvement: LP typically affects the skin, while OLP affects the oral mucosa [18]. Both salivary and serum leptin levels were found to be elevated in patients with OLP compared to healthy controls (Table 3). Additionally, they are elevated in LP compared to healthy control [19]. These findings reflect a systemic inflammatory state in both conditions [19].

However, a significantly higher salivary leptin level was observed in OLP patients compared to LP patients, likely due to the local expression and accumulation of leptin in the oral cavity, which is the site of active mucosal inflammation in OLP [20].

On the other hand, serum leptin levels did not differ significantly between LP and OLP patients, suggesting that the systemic inflammatory burden is comparable in both diseases [20]. This supports the notion that salivary leptin may serve as a more localized and disease-specific biomarker for OLP compared to serum leptin [20].

Strengths of this study include the use of both serum and salivary leptin measurements, the use of ANCOVA to adjust for confounding age differences, and the use of histologically confirmed cases.

A limitation of this study is the difficulty in finding healthy control subjects of the same age. We also faced challenges in convincing patients to provide blood samples, particularly in the control group.

## Conclusion

Salivary and serum leptin levels in OLP patients were higher than in healthy control subjects, suggesting their potential role in the diagnosis of oral lichen planus.

## Data Availability

The data supporting the study’s conclusions are accessible from the corresponding author upon reasonable request.
